# Insecticide resistance in *Anopheles arabiensis* in Sudan: temporal trends and underlying mechanisms

**DOI:** 10.1186/1756-3305-7-213

**Published:** 2014-05-08

**Authors:** Hiba Abdalla, Craig S Wilding, Luisa Nardini, Patricia Pignatelli, Lizette L Koekemoer, Hilary Ranson, Maureen Coetzee

**Affiliations:** 1 Wits Research Institute for Malaria, Faculty of Health Sciences, University of the Witwatersrand, Johannesburg, South Africa; 2 Vector Control Reference Laboratory, Centre for Opportunistic, Tropical and Hospital Infections, National Institute for Communicable Diseases, NHLS, Sandringham, Johannesburg, South Africa; 3 Vector Biology & Control unit, Blue Nile National Institute for Communicable Disease, Wad Medani, Sudan; 4 Department of Vector Biology, Liverpool School of Tropical Medicine, Pembroke Place, Liverpool, UK; 5 Present address: School of Natural Sciences & Psychology, Liverpool John Moores University, Liverpool, UK

**Keywords:** *Anopheles arabiensis*, Malaria, Insecticide resistance, Mechanisms

## Abstract

**Background:**

Malaria vector control in Sudan relies mainly on indoor residual spraying (IRS) and the use of long lasting insecticide treated bed nets (LLINs). Monitoring insecticide resistance in the main Sudanese malaria vector, *Anopheles arabiensis*, is essential for planning and implementing an effective vector control program in this country.

**Methods:**

WHO susceptibility tests were used to monitor resistance to insecticides from all four WHO-approved classes of insecticide at four sentinel sites in Gezira state over a three year period. Insecticide resistance mechanisms were studied using PCR and microarray analyses.

**Results:**

WHO susceptibility tests showed that *Anopheles arabiensis* from all sites were fully susceptible to bendiocarb and fenitrothion for the duration of the study (2008–2011). However, resistance to DDT and pyrethroids was detected at three sites, with strong seasonal variations evident at all sites. The *1014 F kdr* allele was significantly associated with resistance to pyrethroids and DDT (*P* < 0.001) with extremely high effects sizes (OR > 7 in allelic tests). The *1014S* allele was not detected in any of the populations tested. Microarray analysis of the permethrin-resistant population of *An. arabiensis* from Wad Medani identified a number of metabolic genes that were significantly over-transcribed in the field-collected resistant samples when compared to the susceptible Sudanese *An. arabiensis* Dongola strain. These included *CYP6M2* and *CYP6P3,* two genes previously implicated in pyrethroid resistance in *Anopheles gambiae s.s*, and the epsilon-class glutathione-S-transferase, *GSTe4*.

**Conclusions:**

These data suggest that both target-site mechanisms and metabolic mechanisms play an important role in conferring pyrethroid resistance in *An. arabiensis* from Sudan. Identification in *An. arabiensis* of candidate loci that have been implicated in the resistance phenotype in *An. gambiae* requires further investigation to confirm the role of these genes.

## Background

Sudan, in 2010, accounted for the highest number of malaria cases in the World Health Organization Eastern Mediterranean Region (WHO/EMR), and was responsible for 58% of the total regional malaria cases [1]. The 2007 National Strategic Plan for Malaria in Sudan aimed to reduce morbidity and mortality of malaria by 50% by 2012 (National Malaria Control Programme, unpublished data). The use of long-lasting insecticidal nets (LLINs) and indoor residual spraying (IRS) are the main components of most malaria prevention and control strategies because they are highly effective and have a relatively low cost. Currently there are only four classes of insecticides approved for IRS (pyrethroids, organochlorines, organophosphates and carbamates), and only one class, the pyrethroids, allowed for use on LLINs [2]. Given the importance of insecticide use in malaria vector control, and the continuous development of insecticide resistance in malaria mosquitoes, monitoring the susceptibility of vectors to pyrethroids and the other insecticide classes is essential. Furthermore, it is necessary to identify the resistance mechanisms involved [2,3] in order to formulate evidence-based resistance management strategies.

Malaria vector control in Sudan has a long history [4]. The main vector control interventions include IRS and the use of LLINs, chemical larviciding of water bodies/breeding sites, environmental management and some, albeit limited, biological control. The Sudanese Ministry of Health (MOH) has provided free distribution of LLINs in Gezira state since 2005 [1].

Insecticide resistance is widespread in the major African vectors belonging to the *Anopheles gambiae* complex [5-8] and in particular, resistance to pyrethroids has been shown to hamper malaria control programmes [9]. *Anopheles arabiensis* is the main vector in central Sudan and multiple insecticide resistance has been reported in Sudanese populations of this species. This includes resistance to the organochlorides benzene hexachloride (BHC) and dichlorodiphenyltrichloroethane (DDT) [10], the organophosphate malathion [11] and to various pyrethroids [12].

The two best understood mechanisms of insecticide resistance in mosquitoes are target site insensitivity and metabolic resistance [13]. Target site insensitivity to pyrethroids and DDT is a result of changes in the neuronal voltage-gated sodium ion channel (VGSC). In *An. gambiae s.s.*, two point mutations at amino acid position 1014 of the VGSC have been described, resulting in either a leucine to phenylalanine (*L1014F*) [14], or a leucine to serine (*L1014S*) substitution [15]. Both of these knockdown resistance *(kdr*) mutations have been shown to be associated with DDT and pyrethroid resistance phenotypes [15-17]. In *An. arabiensis*, *kdr* mutations have been found in several widely dispersed locations including Burkina Faso, Tanzania, Uganda and Sudan [12,18-21].

Metabolic resistance is generally associated with three large enzyme families: the cytochrome P450 monooxygenases (CYP450s), carboxyl/cholinesterases (CCEs), and glutathione-S transferases (GSTs) [22]. Little information is available on the metabolic resistance mechanisms present in Sudanese *An. arabiensis* populations although Hemingway [11,23] did implicate a carboxylesterase gene in resistance to malathion. Recently, Nardini *et al.*[21] reported that one P450 gene, *CYP9L1*, showed increased transcription in a Sudanese DDT selected laboratory strain (SENN-DDT) compared to an unselected susceptible strain.

In the present study, we describe the insecticide resistance patterns of *An. arabiensis* populations from central Sudan over a three year period, and report the principle mechanisms responsible for the observed resistance to pyrethroids and DDT. Results from this study will guide the development of effective insecticide resistance management strategies in Sudan.

## Methods

### Study area

This study was carried out in Gezira state in central Sudan. The state is situated in a rich savanna environment. The area has a hot dry summer from April to June with daily temperatures between 32-42°C, and relative humidity of 20%. The rainy season starts in late June and ends in October. Winter is relatively cold and dry and occurs from December to February, with daily temperatures between 15-21°C, and a relative humidity of 30% (Sudan Meteorological Services, 2005, unpublished data).

**Figure 1:**
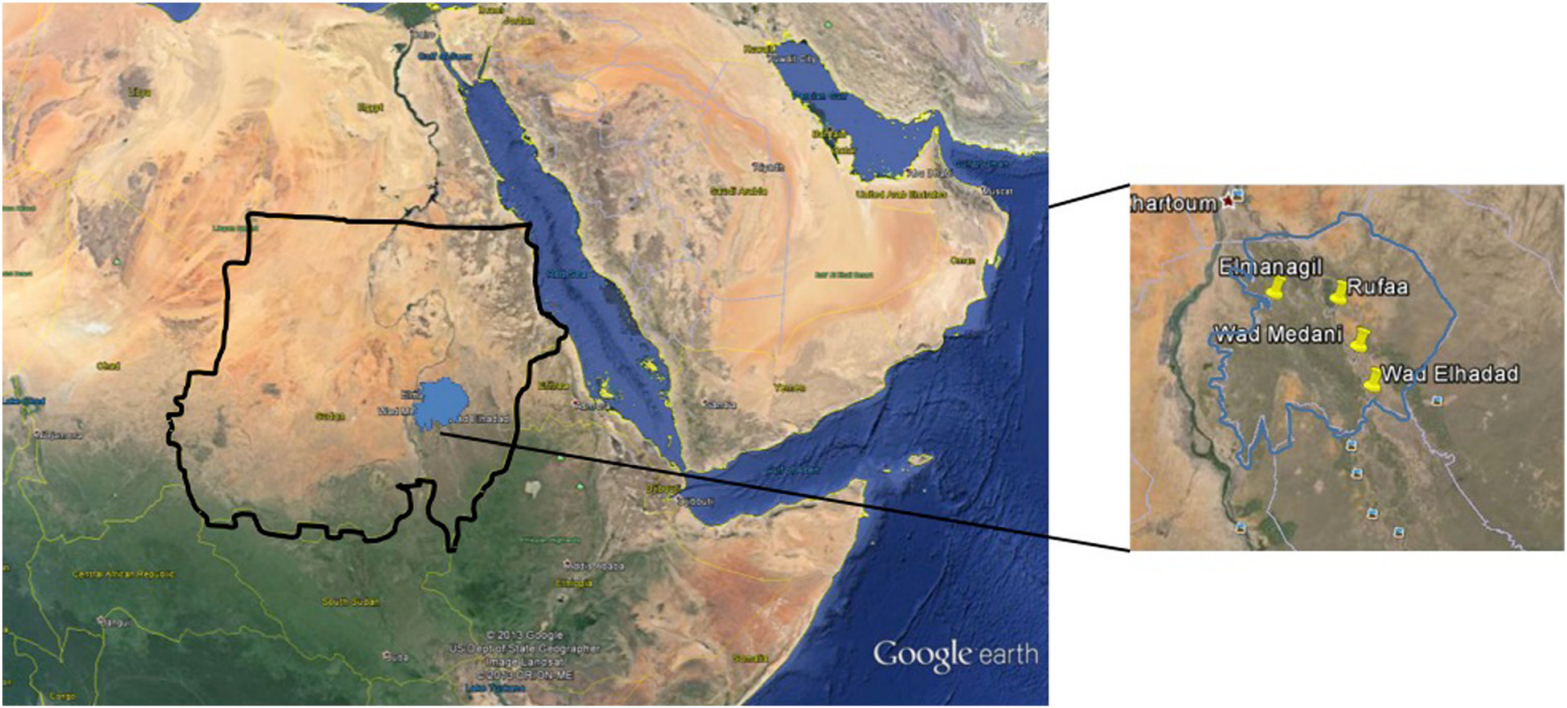
Map showing the location of the study area and the sentinel sites.

Four sentinel sites were identified for biannual resistance monitoring (Figure 1). These sites were chosen to cover a range of insecticide selection pressures. These included an urban site with local use of insecticides in subsistence agriculture (Wad Medani), a rural site with intensive crop cultivation (Elmanagil), a site with very high coverage of insecticide-based malaria control interventions (Wad Elhadad) and a site with no intensive agriculture and no organized vector control programme (Rufaa).

### Mosquito collections

*Anopheles* mosquitoes were collected between October 2008 and January 2011. Two rounds of collections were performed every year in each of the four sites to coincide with the two peaks of malaria transmission seasons (October/November - the period towards the end of the wet season; and January - the dry season). Mosquitoes were collected as larvae and transferred to the BNNICD (Blue Nile National Institute for Communicable Diseases) insectary and reared to the adult stage to be used for WHO insecticide susceptibility tests. Samples were taken from multiple breeding sites within a 10 km radius and collections undertaken over several days to minimize the probability of analyzing siblings from a single female.

### WHO bioassay tests

Larvae were reared to the adult stage, morphologically identified, and only *Anopheles gambiae s.l*. non-blood fed, 3–5 day old adult females were used for the insecticide bioassays. Insecticide papers were obtained from the WHO reference centre in Malaysia. Each batch was tested on the insecticide susceptible Kisumu strain of *An. gambiae* at the Liverpool School of Tropical Medicine before dispatch to Sudan. Five insecticides were tested: 4% DDT (organochlorine), 0.75% permethrin (type I pyrethroid), 0.05% deltamethrin (type II pyrethroid), 0.1% bendiocarb (carbamate) and 1% fenitrothion (organophosphate) according to standard WHO protocols [24].

### Mosquito identification and *kdr* mutation detection

From each sentinel site and for each insecticide, 30–50 mosquitoes taken from the control exposure as well as 20 specimens that survived exposure and 20 specimens that died, were identified to species level using PCR [25]. The hydrolysis probe/Taqman®-*kdr* assay [26] was used for the detection of the *L1014F* and *L1014S* mutations from pyrethroid/DDT bioassay survivors and dead specimens.

### Data analysis

Susceptibility status of mosquito populations was evaluated according to WHO criteria [2]. Mortality greater than 98% indicates susceptibility, mortality less than 98% suggests resistance and needs further investigation, and mortality of 90% or lower confirms insecticide resistance. Mortalities in the control groups were less than 5%, thus no corrections using Abbott’s formula were required [27]. A two sample *t*-test was used to compare the differences in mortalities between the two different seasons during each year of collection and to check for significant change in mortalities over the three years. Analyses were undertaken for each insecticide using Statistix7 analytical software. Regression analysis was used to compare the *kdr* allele frequencies over the three years. Allelic association between *kdr* and resistance, and odds ratios, were calculated in Vassar Stats (http://vassarstats.net/odds2x2.html).

### Microarray analysis

Microarray analysis was performed to test for gene transcription differences between three sample conditions: Wad Medani resistant mosquitoes (survived 120 minute exposure to permethrin, which is equivalent to an LT_80_), Wad Medani unexposed, and the control DONG (susceptible *An. arabiensis* strain from Dongola in North Sudan, susceptible to all known insecticides; housed at BNNICD, Sudan and the Botha de Meillon Insectary, National Institute for Communicable Diseases (NICD), South Africa).

For each biological condition, total RNA was extracted from 5 pools of 15, 3 day old unfed female mosquitoes using the Arcturus PicoPure Kit (Life Technologies, Paisley, UK) with an on-column DNase digestion (RNase-Free DNase , Qiagen, UK). Total RNA concentration was measured using a NanoDrop spectrophotometer (NanoDrop Technologies, Wilmington, USA) and quality assessed using the Agilent RNA 6000 Nano Assay on an Agilent 2100 Bioanalyzer (Agilent Technologies, Stockport, UK). RNA pools (100 ng total) were labeled separately with Cy3 and/or Cy5 using the Agilent Low Input Quick Amp Labeling Kit (Agilent Technologies) following the manufacturer’s recommendations. Labeled RNA was purified using the RNeasy Mini Kit (Qiagen), eluted in 30 μl water, and quantity and quality determined as above. All samples passed the yield and specific activity thresholds recommended by Agilent. Cy3-labelled cRNA (300 ng) and Cy5-labelled cRNA (300 ng) were combined and hybridised to the AGAM-15 K chip [28].

The experimental design for microarray comparisons is depicted in Additional file 1: Figure S1. Hybridization, washing, scanning and feature extraction were performed according to the manufacturer’s recommendations. Analysis was undertaken in GeneSpring GX 11 (Agilent Technologies) following filtering out of all probes where either red or green signal was not significantly above background. A 2-fold cut-off with probability ≤0.05 (FDR adjusted; [29]) was applied to all features in order to determine significance. All data have been submitted to ArrayExpress (http://www.ebi.ac.uk/miamexpress/) with accession number E-MEXP-3656.

### qPCR validation

The microarray results were validated by quantitative real-time PCR (qPCR).

Fold-change differences between Wad Medani resistant and Dongola (susceptible) were calculated for *CYP6P3*, *CYP6Z3* and *GSTe4* with normalization to the control ribosomal reference gene *RSP7*. Primer sequences are provided in Table 1.

**Table 1:** **Primer sequences used for qPCR validation of *CYP6P3*, *CYP6Z3 *** **and *GSTe4***

***Gene***	**Primer name**	**Primer sequence**
*CYP6P3*	P3qf3	5′GTGATTGACGAAACCCTTCGGAAGT3′
	P3r3	5′GCACCAGTGTTCGCTTCGGGA3′
*CYP6Z3*	Z3qF2	5′TGGTCCACGCAATTGCATTGGTCTT3′
	Z3qR2	5′CGCGCATGGGGAAACCATCT3′
*GSTe4*	E4A	5′CCGGTTCCGGTTCTATTTGGAACC3′
	E4B	5′GCCCGATTCGTTGCCTCGTAG3′
*RSP7*	S7-F	TTACTGCTGTGTACGATGCC
	S7-R	GATGGTGGTCTGCTGGTT

Superscript III (Invitrogen) was used to produce cDNA for qPCR. All cDNA samples were cleaned using the PCR Purification Kit (Qiagen) prior to use. A 2-fold dilution series of template cDNA was used to produce the required standard curves. All reactions were prepared in triplicate in 20 μl volumes containing 1× Agilent Brilliant III SYBR qPCR Mastermix, 300 nM of each primer, and 1 μl template RNA (100 ng/ul). Reactions were performed using the Agilent MX3005 qPCR system. The cycling conditions were 3 min at 95°C followed by 10s at 95°C, 10s at 60°C (40 cycles). The ΔΔC_t_ method was used for calculation of fold change values [30].

## Results

### WHO bioassay tests

**Figure 2:**
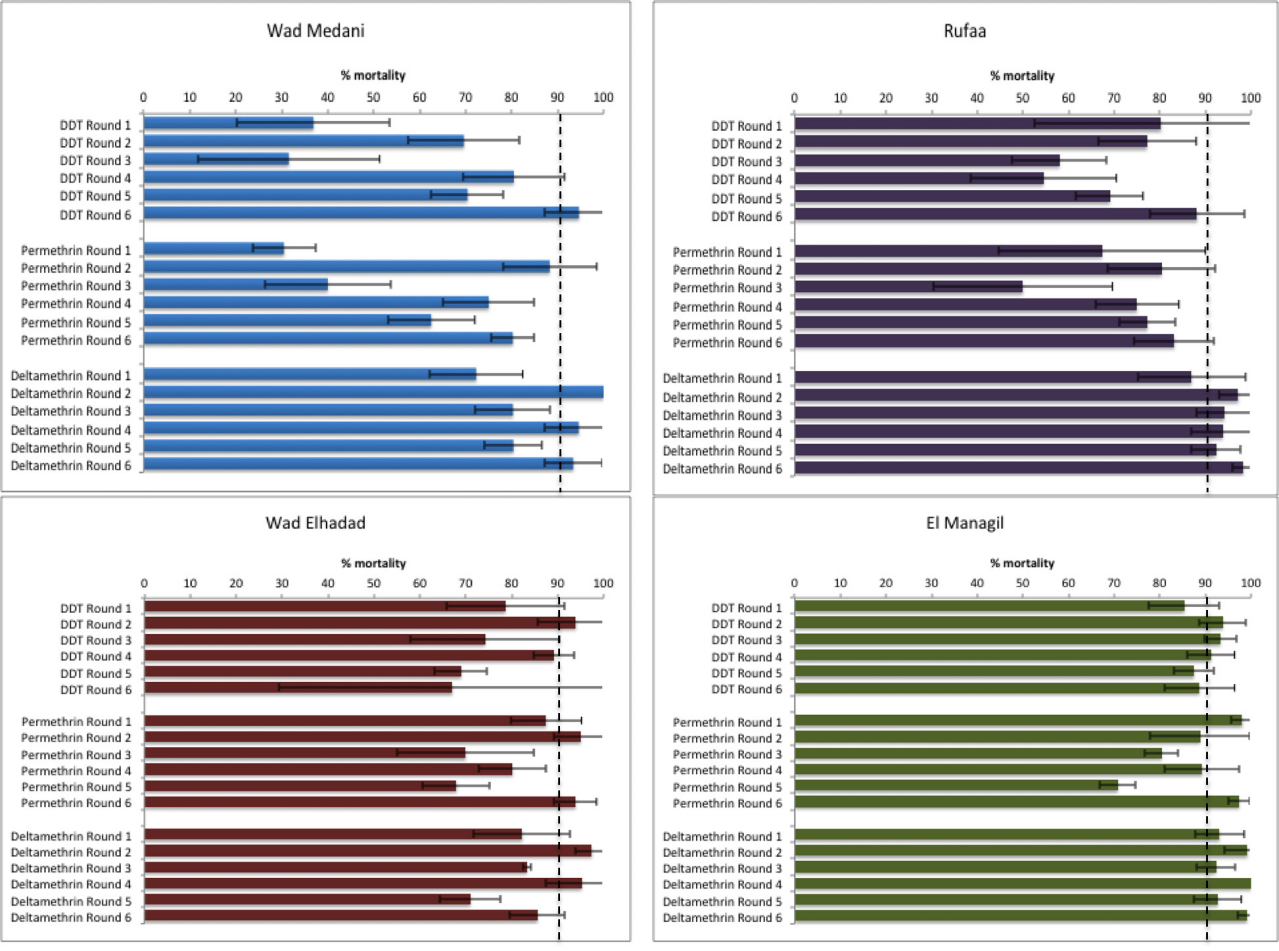
**Insecticide susceptibility status of *Anopheles gambiae s.l*. in Gezira state central Sudan from four sentinel sites using permethrin, deltamethrin, and DDT over three years (2008 – 2011).** Error bars are standard deviations. Dashed line equals 90% cut off for resistance set by WHO (WHO, 2013).

A total of six collection rounds were conducted at four sentinel sites in central Sudan from October 2008 to January 2011 (two rounds per year). During each year of collection, around 2, 270 adult mosquitoes reared from larval collections were exposed (400–450 per insecticide) to five insecticides (permethrin, deltamethrin, DDT, bendiocarb and fenitrothion). Figure 2 shows the susceptibility/resistance status of *An. gambiae s.l.* in these collections. Identification to species level revealed only the presence of *An. arabiensis* from all four sentinel sites. They were fully susceptible to bendiocarb (carbamate) and fenitrothion (organophosphate) at all four sites (data not shown).

High frequencies of permethrin and DDT resistance were observed over the three years of monitoring in the urban agricultural area of Wad Medani, and in Rufaa, the area with low insecticide exposure (Figure 2).

Two sample *t*-tests were used to analyse the differences in susceptibility between the different seasons for each insecticide for each of the three years. Tables 2 and 3 summarize the results of comparisons between seasons within sites, and between years within sites respectively. Seasonal variations were most pronounced in Wad Medani with resistance more pronounced in the wet season than the dry.

**Table 2:** Comparison of the mortalities obtained for each insecticide between the two seasons for each year of monitoring

			**P value**			
**Insecticide**	**Year**	**Season**	**Wad Medani**	**Rufaa**	**Wad Elhadad**	**Elmanagil**
Permethrin 0.75%	Year 1	Oct. –Nov Vs Jan.	**0.0000**	0.2857	0.1241	**0.0369**
	Year 2	Oct. –Nov. Vs Jan.	**0.0016**	**0.0290**	0.2109	0.1130
	Year 3	Oct. –Nov. 2008Vs Jan. 2009	**0.0060**	0.2677	**0.0002**	0.9588
Deltamethrin 0.05%	Year 1	Oct. –Nov. 2008Vs Jan. 2009	0.001	0.3190	0.0284	0.0628
	Year 2	Oct. –Nov. 2008 Vs Jan. 2009	**0.0187**	0.9813	**0.0258**	**0.001**
	Year 3	Oct. –Nov. 2008 Vs Jan. 2009	**0.0107**	0.0548	**0.0063**	0.0819
DDT 4%	Year 1	Oct. –Nov. 2008 Vs Jan. 2009	0.4931	**0.0078**	**0.0369**	0.1688
	Year 2	Oct. –Nov. 2008 Vs Jan. 2009	0**.0012**	0.7006	0.1130	0.4494
	Year 3	Oct. –Nov. 2008 Vs Jan. 2009	**0.0010**	**0.0130**	0.9588	0.2965

*P* values below 0.05 indicate significant difference. (shown in bold).

**Table 3:** Comparison of the mortalities obtained for each insecticide between different year one and three of the study

**Insecticide**	**Year**	**Season**	**Wad Medani**	**Rufaa**	**Wad Elhadad**	**Elmanagil**
Permethrin 0.75%	Year 1 Vs Year 3	Oct. –Nov 2009 VS Oct. –Nov 2010	**0.0000**	0.3324	**0.0034**	**0.0000**
Deltamethrin 0.05%	Year 1 Vs Year 3	Oct. –Nov 2009 VS Oct. –Nov 2010	0.1595	0.2327	0.0754	0.8988
DDT 4%	Year 1 Vs Year 3	Oct. –Nov 2009 VS Oct. –Nov 2010	**0.0060**	**0.0146**	0.1157	0.9963

*P* values below 0.05 indicate significant difference (shown in bold).

### Detection of *kdr*

The *1014 F* allele was significantly associated with resistance to pyrethroids and DDT in all seasons (*P* < 0.001) with effect sizes (odds ratios) ranging from 7.8 to 15.5 in allelic tests (Table 4 and Additional file 2: Table S1). The *L1014S* mutation was not detected in any specimens.

**Table 4:** **Summary of *L1014F kdr *** **genotypes and allele frequencies in alive and dead *An. arabiensis *** **exposed to pyrethroid and DDT from all sentinel sites in central Sudan during all 3 years of collection**

**Insecticide**	**Phenotype**	***Kdr *** **genotype**				**Number of alleles**		**Allele frequency**		**Association test**			
		**FF**	**LF**	**LL**	**Total**	**F**	**L**	**Freq. (F)**	**Freq. (L)**	**Pearson χ**^**2**^	**OR**	**LCI**	**UCI**
Permethrin	Survivors	272	89	30	391	633	149	0.81	0.19	<.0001	15.54	12.26	19.69
	Dead	23	155	290	468	201	735	0.21	0.79				
Deltamethrin	Survivors	143	68	6	217	354	80	0.82	0.18	<.0001	12.99	9.79	17.23
	Dead	32	180	268	480	244	716	0.25	0.75				
DDT	Survivors	149	165	38	352	463	241	0.66	0.34	<.0001	7.81	6.25	9.74
	Dead	13	166	307	486	192	780	0.20	0.80				

OR = odds ratio for association of *1014 F* with resistance. LCI/UCI = lower and upper C.I.s of OR.

### Microarray analysis

No significantly differentially expressed genes were detected between the Wad Medani population that survived exposure to the permethrin LT_80_ and theWad Medani unexposed controls (Additional file 3: Table S2). However, in comparisons of Wad Medani resistant samples with the DONG susceptible strain 1,341 probes showed differential transcription levels (FC ≥ 2; *P* < 0.05; Additional file 3: Table S2 and Additional file 4: Table S3). The lowest *p* values were for unidentified proteins followed by trypsins and chymotrypsins (Additional file 4: Table S3). However, numerous detoxification genes were over expressed in the permethrin resistant field population compared to the laboratory susceptible colony (Table 5). Genes from the three families commonly associated with insecticide metabolism are each represented by 4 probes on the array and genes for which all four probes were significant, and whose maximum fold change was greater than 5 were selected as the strongest candidates. This candidate list included *CYP6Z3, CYP6M2, CYP6P3, and GSTe4.*

**Table 5:** **Significantly up-regulated (*p*** **< 0.05; FC > 2) detoxification genes in whole genome microarray comparison of Wad Medani resistant *vs*. Dongola colony**

**Gene**	**Vectorbase descriptor**	**FC min**	**FC max**	**N° probes**
**Cytochrome P450s**				
*CYP6Z3*	AGAP008217-RA	7.97	13.45	4
*CYP6P3*	AGAP002865-RA	6.73	14.99	4
*CYP6M2*	AGAP008212-RA	4.82	5.03	4
*CYP4H24*	AGAP013490-RA	4.50		1
*CYP6P4*	AGAP002867-RA	3.93	4.36	4
*CYP6Z2*	AGAP008218-RA	3.57	4.38	3
*CYP6AG1*	AGAP013511-RA	3.08	3.14	2
*CYP6M3*	AGAP008213-RA	2.80	2.82	3
*CYP302A1*	AGAP005992-RA	2.73		1
*CYP9J5*	AGAP012296-RA	2.67	2.71	4
*CYP9K1*	AGAP000818-RA	2.37		1
*CYP4H19*	AGAP000088-RA	2.32	4.37	4
*CYP9L2*	AGAP012850-RA	2.10		1
*CYP4C28*	AGAP010414-RA	2.05	2.97	4
*CYP12F2*	AGAP008021-RA	2.00	2.42	4
*CYP6AK1*	AGAP010961-RA	2.03		1
*CYP325J1*	AGAP001443-RA	3.72	3.81	3
**Carboxylesterases**				
carboxylesterase	AGAP010912-RA	6.94		1
*COEglt2H*	AGAP010914-RA	6.68		1
*COEJHE2E*	AGAP005834-RA	3.06	4.01	3
*COEJHE5E*	AGAP005837-RA	4.65	10.22	2
*COEAE2D*	AGAP005757-RA	2.01	2.07	3
*COE09941* - Putative Carboxylesterase	AGAP010915-RA	2.72	2.89	3
*COE13O*	AGAP011507-RA	2.34	2.58	4
*COE09895* - Putative Carboxylesterase	AGAP010911-RA	2.06	2.26	4
**Glutathione-S-transferases**				
*GSTe4*	AGAP009193-RA	15.60	23.31	4
*GSTd1-5*	AGAP004164-RA	2.24	2.29	4
*GSTd3*	AGAP004382-RA	2.23	2.50	4

N° probes = number of significant probes for each gene on the microarray (maximum = 4).

### qPCR analysis

qPCR validated the microarray results for *CYP6P3* (microarray FC = 12.45; qPCR FC = 13.35) and for *GSTe4* (microarray FC = 2.41; qPCR FC = 3.2). For *CYP6Z3*, the FC value obtained from microarray analysis (10.39) was lower than that obtained using qPCR (21.35) but was still significant.

## Discussion

Previous studies have shown that insecticide resistance is present in Sudanese vector populations [10-12,23,31,32]. The present study extends this work through evaluation of the resistance status of *An. arabiensis* over three years of monitoring in areas with different levels of insecticide exposure and explores the difference in resistance patterns between the two different seasons of malaria transmission.

*Anopheles arabiensis* was the only member of the *An. gambiae* complex found in Gezira state, as has been demonstrated previously [4,12,32,33]. The field population of *An. arabiensis* collected from Gezira state was susceptible to bendiocarb (a carbamate) and fenitrothion (organophosphate). Bendiocarb is currently used for IRS in Gezira state. It was first applied for public health purposes in central Sudan in 2007 when *An. arabiensis* showed strong resistance to the pyrethroid insecticide, permethrin [12].

*Anopheles arabiensis* from central Sudan exhibited resistance to permethrin, DDT and deltamethrin. The most resistant population was found in Wad Medani, an urban agricultural site. The presence of multiple insecticide resistance in the study area is consistent with previous studies [12,31,32]. There is strong variation in mortality rates between the two different seasons of each year of collection (wet/dry). For example, in Wad Medani, permethrin and DDT resistance during the 2008–2009 rounds of collections was high with mortality rates of 33% and 35.5% respectively during the wet season. During the dry season, the mortality rates increased to 89% and 73.4% for permethrin and DDT respectively. The same trend is observed for deltamethrin where the mortality was 73.2% during the wet season and 100% during the dry season. Such seasonal variations in susceptibility of *Anopheles* mosquitoes to insecticides have been reported in Burkina Faso [34], Benin [35] and Chad [36].

Resistance in the mosquito population to permethrin, and the reduced susceptibility to deltamethrin in Wad Elhadad, a site of intensive LLIN coverage, may have developed as a result of sustained exposure of adult mosquitoes to these insecticides from LLINs. DDT is currently not used in Sudan, however, high levels of resistance were still observed. DDT resistance was strongly associated with the *L1014F* mutation and it is possible that recent pyrethroid use has selected for this cross-resistance mechanism. As a result, high levels of DDT resistance was observed, even in areas where DDT use had been discontinued for a number of years. Similar results were reported in the same area in previous studies [12,32]. The *kdr* allele *1014 F* is strongly associated with resistance in these populations. Effects sizes (Odds Ratios) are high; in allelic tests they are similar to the values reported previously for effects sizes of *kdr* in Ghanaian and Cameroonian *An. gambiae*[37].

While *1014 F* imparts a dramatic effect on resistance, it does not completely explain the resistance phenotype since many *1014 L* homozygotes survive 1 hr exposure to pyrethroids and DDT, suggesting that alternative mechanisms of resistance are operating in this population. This phenomenon has been described before [21,38,39].

In order to investigate the metabolic resistance mechanisms involved in the resistance of these field populations, a whole genome microarray approach was used. In comparisons of *An. arabiensis* from Wad Medani, which survived exposure to permethrin equivalent to an LT_80_, with the susceptible DONG strain, a large number of genes showed significantly different transcription levels. The most significantly over-transcribed genes were trypsins, chymotrypsins and loci of no known function for which the relationship with resistance is difficult to investigate. A number of detoxification genes were also significantly over-transcribed, including genes with known roles in permethrin metabolism, e.g. *CYP6P3* and *CYP6M2*, both of which have been demonstrated to be capable of metabolising pyrethroids *in vitro*[40,41]. In addition to this, *CYP6M2* was recently shown to also metabolise DDT [28]. The highest over-transcribed candidate was the glutathione-S-transferase *GSTe4* which exhibits >15 fold mRNA expression in the resistant strain. Whilst GSTs have recognised roles in DDT metabolism, they may also be associated with pyrethroid resistance [42].

No significantly over-transcribed probes were detected in comparisons of Wad Medani resistant samples with unexposed controls from the same site. However, this may be explained by significant effects of *1014 F* on resistance since samples for microarray were not matched for *kdr* status. In addition, there is reduced power to detect gene expression differences in comparisons of resistant samples with unexposed controls since these, by definition, will contain resistant individuals. The high levels of resistance and the importance of both target-site and metabolic processes in the resistance phenotype indicate that continued monitoring of expression of detoxification loci in resistant populations of *An. arabiensis* is necessary.

Whether the resistance found in central Sudan is due to agricultural usage of chemicals, domestic aerosols/coils/ITNs for personal protection or from malaria vector control activities is difficult to assess as we do not have data on the types or quantities of chemicals used for agriculture or domestic use. What is clear, however, is that resistance has been found at all four study sites, albeit at different frequencies. This endorses the malaria vector control programme’s implementation of a resistance management strategy for central Sudan, as mentioned above, along the lines advocated by the WHO Global Plan for Insecticide Resistance Management [2].

## Conclusions

This study reports strong seasonal variation in the susceptibility levels to pyrethroids and DDT in Gezira state. The results obtained in this study will enable informed choice of insecticides for use in vector control programmes in Gezira state. In addition, the data obtained will provide baseline information needed in the monitoring of the susceptibility of *An. arabiensis* to the carbamate insecticide bendiocarb currently being used by the vector control programme for indoor residual house spraying.

## Competing interests

The authors declare that they have no competing interests.

## Authors’ contributions

HA, CSW, LN and PP were responsible for the field work and laboratory processing of material; LLK, HR and MC designed the project. All authors contributed to the data analysis, drafting the manuscript and all approved the final version.

## Supplementary Material

Additional file 1: Figure S1 Microarray design. Arrows indicate details of labelling: Cy3 → Cy5. N = Wad Medani non exposed controls. R = Wad Medani resistant to permethrin. D = DONG susceptible strain. Click here for file

Additional file 2: Table S1 Summary of L1014F *kdr* genotypes and allele frequencies in alive and dead *An. arabiensis* exposed to pyrethroid and DDT from four sentinel sites in central Sudan during six rounds of collections over three years. Click here for file

Additional file 3: Table S2 Summary of significantly differentially expressed probes. The total number of probes following filtering out of probes not significantly > background is also given. Click here for file

Additional file 4: Table S3 List of genes, with corrected *p*-values and Fold Change, significantly differentially expressed in Wad Medani vs Dongola arrays. Click here for file
