# Supplementary figures and images for: Insecticide resistance in Anopheles arabiensis in Sudan: temporal trends and underlying mechanisms
Source: Parasit Vectors. 2014 May 8;7:213. doi: 10.1186/1756-3305-7-213 (PMC4026821; doi:10.1186/1756-3305-7-213)

## Slide 1
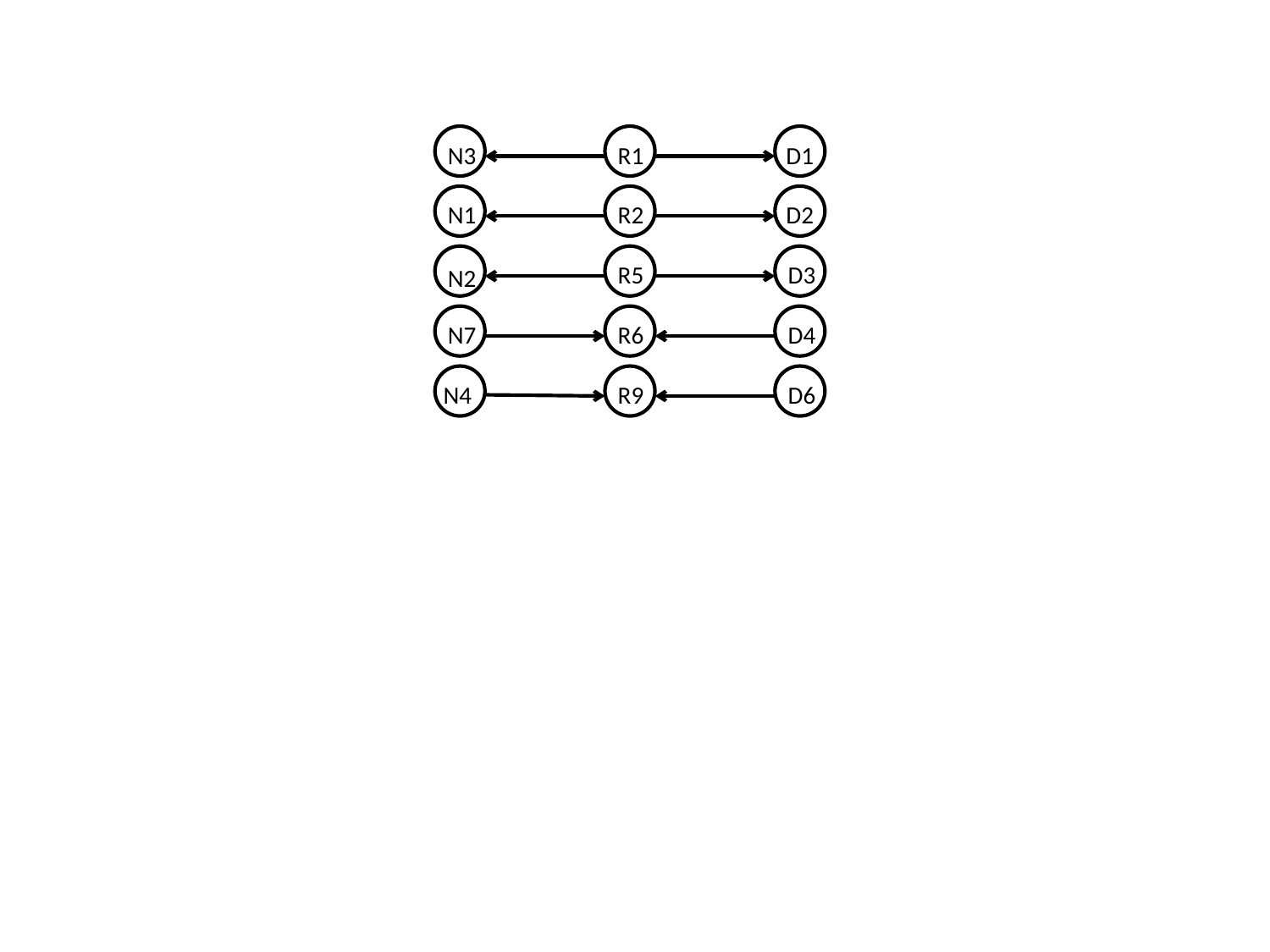

N3
R1
D1
N1
R2
D2
R5
D3
N2
N7
R6
D4
N4
R9
D6

Supplement: Additional file 1: Figure S1 — Microarray design. Arrows indicate details of labelling: Cy3 → Cy5. N = Wad Medani non exposed controls. R = Wad Medani resistant to permethrin. D = DONG susceptible strain. [file 1756-3305-7-213-S1.pptx]
