# Supplementary material for: Insecticide resistance in Anopheles arabiensis in Sudan: temporal trends and underlying mechanisms
Source: Parasit Vectors. 2014 May 8;7:213. doi: 10.1186/1756-3305-7-213 (PMC4026821; doi:10.1186/1756-3305-7-213)
Supplement: Additional file 2: Table S1 — Summary of L1014F kdr genotypes and allele frequencies in alive and dead An. arabiensis exposed to pyrethroid and DDT from four sentinel sites in central Sudan during six rounds of collections over three years. [file 1756-3305-7-213-S2.doc]

**Table S1** Summary of L1014F *kdr* genotypes and allele frequencies in alive and dead *An. arabiensis* exposed to pyrethroid and DDT from four sentinel sites in central Sudan during six rounds of collections over three years
