# Supplementary material for: Insecticide resistance in Anopheles arabiensis in Sudan: temporal trends and underlying mechanisms
Source: Parasit Vectors. 2014 May 8;7:213. doi: 10.1186/1756-3305-7-213 (PMC4026821; doi:10.1186/1756-3305-7-213)
Supplement: Additional file 3: Table S2 — Summary of significantly differentially expressed probes. The total number of probes following filtering out of probes not significantly > background is also given. [file 1756-3305-7-213-S3.docx]

**Table S2*.*** Summary of significantly differentially expressed probes. The total number of probes following filtering out of probes not significantly > background is also given.

|  | Fold Change | *P*< 0.05 | *P*< 0.01 | *P*< 0.001 |
| --- | --- | --- | --- | --- |
| RES *vs*. DONGOLA (12,763 probes) | FC > 2 | 1341 | 358 | 58 |
|  | FC > 3 | 617 | 232 | 42 |
| RES *vs*. NON-EXPOSED (18,086 probes) | FC > 2 | 0 | 0 | 0 |
